# To make people save energy tell them *what* others do but also *who* they are: a preliminary study

**DOI:** 10.3389/fpsyg.2015.01287

**Published:** 2015-08-28

**Authors:** Michele Graffeo, Ilana Ritov, Nicolao Bonini, Constantinos Hadjichristidis

**Affiliations:** ^1^Department of Information Engineering and Computer Science, University of TrentoTrento, Italy; ^2^Department of School of Education and Center for Rationality, Hebrew UniversityJerusalem, Israel; ^3^Department of Economics and Management, University of TrentoTrento, Italy; ^4^Research Centre for Decision Making, Leeds Business School, Leeds UniversityLeeds, UK

**Keywords:** social norms, comparative feedback, nudge, identified victim effect, pro-environmental behavior

## Abstract

A way to make people save energy is by informing them that “comparable others” save more. We investigated whether, one can further improve this nudge by manipulating *Who* the “comparable others” are. We asked participants to imagine receiving feedback stating that their energy consumption exceeded that of “comparable others” by 10%. We varied *Who* the “comparable others” were in a 2 × 2 design: they were a household that was located either in the same neighborhood as themselves or in a different neighborhood, and its members were either identified (by names and a photograph) or unidentified. We also included two control conditions: one where no feedback was provided, and one where only statistical feedback was provided (feedback about an *average* household). We found that it matters *Who* the “comparable others” are. The most effective feedback was when the referent household was from the same neighborhood as the individual's and its members were not identified.

## Introduction

One way to achieve a cleaner, healthier environment is by investing in green technologies such as smart lamps, solar cells, and electric cars. Developing such technologies is costly, but their cost is eventually offset by environmental benefits. A complementary way is by persuading citizens to conserve energy. But how can this be achieved? Psychological research suggests that there are two routes to persuasion, a “central route” that appeals to people's minds and a “peripheral route” that appeals to people's gut instincts (Petty and Cacioppo, 1981). A particularly effective way to persuade people to conserve energy is by informing them that “comparable others” consume less (see Ferguson et al., 2011; Rabinovich et al., 2012). A study on towel reuse in hotels, for example, compared the effectiveness of the sign “JOIN YOUR FELLOW GUESTS IN HELPING TO SAVE THE ENVIRONMENT” followed by the indication that 75% of other guests in that room reused their towels against the standard sign “HELP SAVE THE ENVIRONMENT” (Goldstein et al., 2008, Exp. 1). The first sign triggered a towel reuse rate of 44.1% against the standard sign's 35.1%. Interestingly, people do not realize the influence that norms have on their behavior (e.g., Nolan et al., 2008), suggesting that these operate through the peripheral route. Importantly, such interventions are easy to implement—it suffices to place doorhangers with the appropriate message in people's homes or hotel rooms—and come at a low cost.

Here, we ask whether we can further improve people's energy saving behaviors by manipulating *who* the “comparable others” are. We asked Israeli students^1^ to imagine receiving a message stating that their energy consumption level exceeded that of a comparable household by 10%. They had to state whether they intended to modify their energy consumption (Yes/No) and, if yes, by what amount. We manipulated the referent household along two dimensions in a 2 × 2 design: (1) *Social distance*: the household was located in the participant's neighborhood (in-group) vs. in a different neighborhood (out-group); (2) *Identification*: its members were identified by name, age, and a photograph (identified) vs. they were presented in an abstract way (unidentified). Following research, which we will unpack below, we expected to observe the highest intention to reduce energy consumption when the referent group was from the same neighborhood and identified (Identified—In-group combination).

The introduction proceeds as follows. First, we present additional research showing that messages of what most others *do* (“descriptive norms”) and/or what most others *should do* (“injunctive norms”) promote energy saving behavior. Next, we focus on research suggesting that people are more willing to comply with a request to help in-group members rather than out-group members and identified rather than unidentified individuals. Then, we combine these lines of research and present the current hypothesis.

## The role of social norms in promoting energy consumption

In a clever field study, the littering behavior of people returning to pick their cars from a parking lot was monitored (Cialdini et al., 1990). The experimenters positioned a large handbill under each car's windshield wiper and in alternate times they manipulated, how clean the parking lot was (very clean vs. heavily littered). The variable of interest was how often the subjects littered (threw the handbill on the parking floor) in each condition. Perhaps unsurprisingly, subjects were less likely to litter when the parking lot was clean than when it was dirty. In the same study, the experimenters also manipulated the extent to which subjects' attention was focused on the parking floor. A confederate walked in the direction of the subject holding a handbill. In some occasions, the confederate threw the handbill on the parking floor when in close proximity to the subject whereas, in others occasions the confederate walked by the subject without littering. Interestingly, people were least likely to litter when the confederate littered an otherwise clean parking floor and most likely to litter when the confederate littered a heavily littered parking lot. The idea is that the act of littering drew the subjects attention on the parking floor activating the appropriate descriptive norm: most others do not litter (clean parking floor) or most others do litter (heavily littered parking floor). The authors also discussed the possibility that a clean parking floor might instead activate an injunctive norm, i.e., that people ought to keep the parking lot clean (for another study on the role of injunctive norms, see Hilton et al., 2014).

Social norms do not require direct observation, but can also be triggered through printed messages about what others are doing (for a recent meta-analysis on the effectiveness of various techniques of social influence including social norms and comparative social feedback, see Abrahamse and Steg, 2013). In an ingenious field study on energy consumption, the experimenter team left messages in doorhangers at people's homes (Schultz et al., 2007). The messages reported whether the household's consumption level was below or above that of the average household. The effectiveness of these messages was measured against real meter readings before and after the intervention. Consumers that received negative feedback consumed less in the next period. However, consumers that received positive feedback consumed more in the following period (this is known as the “boomerang” effect). The message is clear: People make adjustments in the direction of the descriptive norm. In a follow up study, the authors found a way to beat the boomerang effect. Together, with the normative feedback they included an emoticon—a happy face for low-consumers or a frowning face for high-consumers—which communicated what people should be doing. With the emoticons in place, not only did the high-consumers consume less but also the low-consumers stayed low!

## The role of social distance and identification

The main experimental goal of the present study was to link the literature on the identified victim effect with literature on the influence of social norms. Specifically, we investigated how the social distance from the referent group (in-group vs. out-group) and the level of identification of the referent group (identified vs. unidentified) combine to influence energy saving behavior. Because, as far as we know, there are no studies that have addressed the interactive effect of these factors on energy saving (but see last paragraph of this section), we develop our hypothesis by focusing on research in another domain, generosity. Generosity is linked to norm adherence—being generous to others can be seen as adhering to a social norm about helping others.

People treat others differently (mostly better) when they belong to their in-group as opposed to their out-group. Numerous studies show preferential treatment and greater generosity toward member of one's own group. People also treat others differently (mostly better) when these are identified rather than unidentified (Schelling, 1968). For instance, people are more willing to comply with a request to donate money to a person in need when the person is described in detail (identified victim) rather than when the person remains unidentified, a “statistical” victim (Jenni and Loewenstein, 1997; Small and Loewenstein, 2003; Kogut and Ritov, 2005a,b; Small et al., 2006; Slovic, 2007; Cryder and Loewenstein, 2010; Cryder et al., 2013). Importantly, studies suggest that these factors interact. Kogut and Ritov (2007), for example, found that willingness to comply with a request to donate in favor of a single identified individual is greater than willingness to help a group of individuals, but only when the perceivers regard the victims as belonging to their own in-group: identifying tsunami victims by name increased actual contributions only when the specified target was a single compatriot.

This effect was also demonstrated in a lab experiment with randomly generated groups (Ritov and Kogut, under review, Study 1). Following the classic minimal group paradigm (Tajfel et al., 1971) participants were asked to rank three pictures in terms of aesthetic pleasantness. Next, they were assigned to one of two groups presumably on the basis of their picture ranking (in reality, the experimenters implemented random assignment). Subsequently, participants played a dictator game against a member of either their in-group or their out-group. The dictator game involves two players: a dictator and a receiver. The dictator is endowed with a sum of money (e.g., 20$) and is given the option to allocate part of it to the receiver. Each player gets paid according to the dictator's allocation. Perhaps unsurprisingly, dictators allocated more money to receivers from their in-group than from their out-group. It is noteworthy to mention that economic rationality mandates that dictators should keep all the money for themselves. In this study, the in-group/out-group manipulation was crossed with whether the receiver was identified/unidentified (by the receiver's experimentally assigned number). Overall dictators were more generous to identified rather than to unidentified receivers. Importantly, however, the main effects of Social distance and Identification were qualified by a significant interaction. Dictators were most generous to in-group—identified players, and equally (un)generous to everybody else.

Although these two factors have not been explicitly addressed in energy consumption studies, the authors of the towel reuse study examined the effect of different referent categories (Goldstein et al., 2008; see also Ferguson et al., 2011). In their Experiment 2, the descriptive norm sign that a subject received was attached to one of four categories: fellow guests, fellow guests that stayed in the exact same room as the subject (the room number was provided), fellow citizens, or men and women. The highest towel reuse rate was observed when the descriptive norm was attached to fellow guests that stayed in that exact room (49.3%). The other conditions showed similar towel reuse rates with an average of 42.8%. As a means of examining, the underpinning mechanism of this effect, Goldstein and colleagues asked a separate group of 53 participants to rate how important it was to their identity being a member of the following categories: an environmentally concerned individual, a hotel guest, a citizen, a male or female, or a guest in the particular room in which they were staying. They found that the last category—which promoted the highest norm compliance—was at the bottom of the participants' lists! Once again, this shows that signs containing descriptive norms persuade people via the peripheral route. For the present purposes, note that the category that worked best was highly “identified.”

## Present research and hypothesis

The aim of the present research was to investigate the comparative effectiveness of four different types of interventions on self-rated intentions to conserve energy. All interventions involved a printed message stating that the individual's energy consumption exceeded that of a referent household by 10%. What varied across the interventions was the information regarding the referent household. Motivated by the research reported in the previous section, we varied this information along two dimensions: (1) *Social distance*: whether the referent household was in the same neighborhood as the subject's (in-group) vs. in a different neighborhood (out-group); and (2) *Identification:* whether the individuals of the referent household were identified by name, age, and a photograph (identified) vs. such information was omitted (unidentified). Merging the research on descriptive norms with that on in-group/out-group, and identified/ unidentified, we expected to observe the highest energy saving in the In-group—Identified condition. Furthermore, we included two control conditions (see below), which aimed to act as a baseline. Our purpose was to measure the effectiveness of the four communication strategies against two baselines: one where only statistical feedback is provided and one where no feedback is provided.

Following a traditional line of research in judgment and decision-making (Kahneman and Knetsch, 1992; Kahneman and Ritov, 1994; Kahneman et al., 1998, 2000; Sunstein et al., 2002), the focus of the present study was to examine people's intentions to conserve energy, rather than actual behavior. The present study is the first to examine the combined effect of social distance and identification on people's intentions to conserve energy.

## Study

### Method

The experiment was performed in accordance with the ethical standards laid down by the 1964 Declaration of Helsinki. We followed the relevant guidelines of the Hebrew University of Jerusalem regarding questionnaires on decision making and social psychology experiments. None of our questions collected sensible data, therefore the University tacitly approved the study. Participants were 334 university students living in Jerusalem (216 participants provided demographic details: 58% of them were females; *M*_age_ = 25.4 years old, years, *SD* = 3.17, age range: 20–40), and data were collected over two adjacent semesters. A preliminary analysis shows that the collection period had no influence on the variables of interest so we run all the following analyses on a single set of data. The participants were contacted at the Hebrew University of Jerusalem by a research assistant. The experiment was run in labs and common rooms of the university. In the first collection period, participants were randomly assigned to one of four experimental conditions, which resulted by crossing the Social distance of the referent household with the level of Identification of its members in a 2 × 2 design. The resulting conditions were: In-group—Identified, In-group—Unidentified, Out-group—Identified, and Out-group—Unidentified. In the second collection period, we also included two control conditions, and participants were randomly assigned to one of the six resulting conditions. In what follows, we first present the methods and results concerning the four experimental conditions. Subsequently, we describe the control conditions, and how the results from the experimental conditions compare to those of the control conditions.

### Experimental conditions

**Figure 1:**
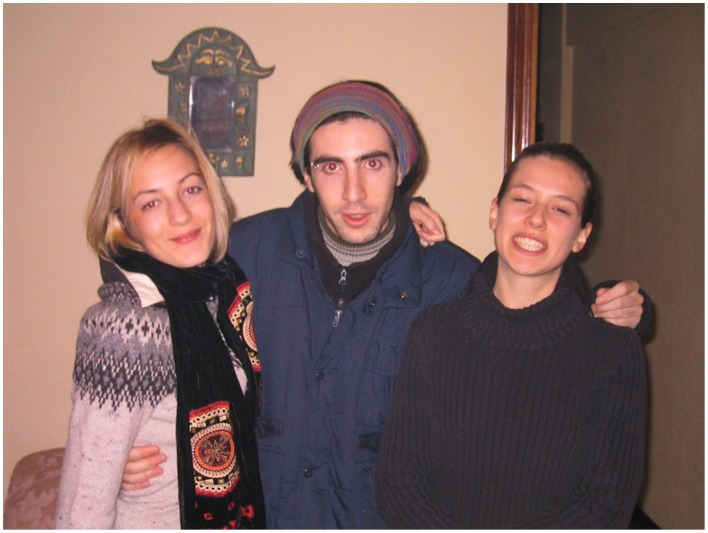
**Picture of the identified referent apartment (participants in the identified conditions also received information about the names and ages of these individuals)**.

In all experimental conditions, participants read a scenario that described the energy consumption behavior of a typical three-student apartment. In the in-group conditions, participants read that the referent apartment was located in their neighborhood (somewhere in Jerusalem), whereas in the out-group conditions that it was located in another city (Haifa). In the identified conditions the name, age, and a photograph of the three students living in the household were provided (see Figure 1), whereas in the unidentified conditions such information was omitted. Participants were then asked to select whether they intended to increase, keep at the same level, or decrease their energy consumption. If they selected to modify their energy consumption, they had to state by how much in terms of a percentage value. We also presented a list of possible means by which the participants could reduce their energy consumption (see below) and we asked them to select three means and rank them in terms of how much they were willing to implement them (i.e., first, second, and third action most likely to be implemented).

Below, we present the instructions used for the In-group—Unidentified condition, followed by the question pertaining to the willingness to modify current consumption level. The original materials were in Hebrew, below we provide the English translation. The instructions for the other conditions are presented in Supplementary Material.

*Imagine that the letter containing your energy bill has arrived. You open it and notice that together with your energy bill there is also a statement comparing your latest consumption level to the average consumption level of a typical apartment from your neighborhood (that is, an apartment where three students live)*.

*The statement notes that: Your energy consumption exceeded the typical apartment consumption in your neighborhood by 10%*.

*In light of this statement, what do you plan to do? Please tick the option that applies below. If you select option 1 or 3, please specify also the appropriate level*.

1. I plan to increase my energy consumption by approximately _____ %
2. *I do not plan to either increase or decrease my energy consumption*.
3. I plan to decrease my energy consumption by approximately _____ %

*If you selected option 3 (decrease your energy consumption level), please specify the means by which you aim to achieve this by ticking up to three statements from the list below. Next to each of these statements, please indicate how much you are willing to actually implement these solutions: “1”* = *most likely to implement; “2”* = *second most likely to implement; “3”* = *third most likely to implement*.

- *Turn off the light when you exit the room*.
- *Substitute the old light bulbs in your house with low consumption ones*.
- *Do the laundry during off-pick hours*.
- *Substitute high consumption electric appliances (e.g. dishwashers, irons) with more energy efficient models*.
- *Air dry dishes instead of using your dishwasher's drying cycle*.
- *Turn off your computer and monitor when not in use*.
- *Wash only full loads of dishes and clothes*.

Following these tasks, participants were asked a series of ancillary questions whose objective was to check the perceived effectiveness of the manipulation: (1) “To what extent do you consider important (for your energy consumption choices) the information given above about the typical apartment?” (7-point response scale from 0 = *Not important at all* to 6 = *Very important*); (2) “To what extent do you feel that the place where you live is similar to the typical apartment in your neighborhood (that is, to an apartment where three students live)?” (7-point response scale ranged from 0 = *Not similar at all* to 6 = *Very similar*); (3) “Including yourself, how many people live in your apartment (answer “1” if you live alone; “2” if you live with just one other person; etc.) _____”; (4) “How does your actual energy consumption level compare to the consumption level of other apartments in your neighborhood that have a similar composition to yours (that is, other apartments with the same number of individuals)?” (7-point response scale ranging from −3 to +3; −3 = *My consumption is much lower*, 0 = *My consumption is similar*, and +3 = *My consumption is much higher*); (5) “In which neighborhood do you live?” Table 1 illustrates the means (*SD*s) of these variables by experimental condition.

**Table 1:** **Mean scores (SDs) of the ancillary variables by Type of Feedback**.

	**Social feedback**			
	**Unidentified**		**Identified**	
	**In-group (*n* = 69)**	**Out-group (*n* = 70)**	**In-group (*n* = 69)**	**Out-group (*n* = 69)**
Mean (*SD*) perceived importance of the information (0–6 scale)	3.23 (1.68)	3.06 (1.37)	3.04 (1.53)	2.67 (1.65)
Mean (*SD*) perceived similarity between participants apartment and the referent apartment (0–6 scale)	2.65 (1.50)	2.73 (1.46)	3.04 (1.33)	3.00 (1.32)
Mean (*SD*) number of people living in the participants' apartment including the participant	2.62 (1.35)	3.31 (1.65)	2.96 (1.34)	2.83 (1.21)
Mean (*SD*) participant's actual energy consumption, compared with their neighbors consumption (−3 to +3 scale)	−0.23 (0.99)	0.11 (1.03)	0.16 (1.21)	0.0 (1.07)

## Results

### Manipulations checks

We first examined, whether the four experimental conditions differed in terms of (a) the perceived importance of the information given and (b) the perceived similarity between the participant's household and that described in their information pack (see Table 1), and (c) the perceived energy consumption level with respect to other apartments from the participant's neighborhood. We examined each dependent variable by means of a 2 (Social distance: in-group vs. out-group) × 2 (Identification: identified vs. unidentified) between-subjects analysis of variance (ANOVA). The perceived importance of the information did not vary significantly across the conditions (all *p*s > 0.12). Overall, the participants considered the description of the household as quite important, with many answers concentrated on the central value of the 0–6 scale (*M* = 3, *SD* = 1.57). The perceived similarity varied significantly across experimental conditions: Participants rated themselves as marginally more similar to the people described in the identified conditions than to those mentioned in the unidentified conditions (*M*_Identified_ = 3.02 vs. *M*_Unidentified_ = 2.69), *F*_(1, 273)_ = 3.84, *p* = 0.051, η_*p*_^2^ = 0.01. No differences were found among the experimental conditions in terms of the perceived energy consumption level of the participant's apartment with respect to other apartments from their neighborhood.

Finally, we controlled some further aspects of our experimental manipulation. Firstly, we checked whether our description of a three-student apartment was a realistic reference point by asking how many people live in the actual apartment of the participants. It was. The mean number of persons living in the participants' apartments was very close to 3 (*M* = 2.94; *SD* = 1.40). Secondly, we controlled where the participants lived. None lived in Haifa, and so the Out-group referent was correctly named “out-group.”

### Self-rated intention to modify consumption: Choice

**Figure 2:**
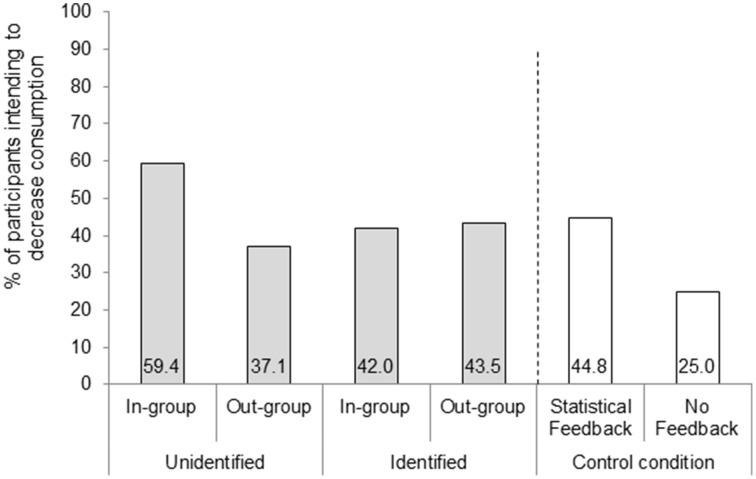
**Percentage of participants intending to decrease energy consumption by condition**. The number of participants in each group were as follows: In-Group Unidentified (*n* = 69), Out-Group Unidentified (*n* = 70), In-Group Identified (*n* = 69), Out-group Identified (*n* = 69), Statistical Feedback (*n* = 29), and No Feedback (*n* = 28).

We then turned to the participants' energy consumption choices. A preliminary inspection revealed that all participants selected to either decrease or leave unmodified their current energy consumption—no one decided to increase it (option 1). We thus coded their choices by means of a binary variable: decrease consumption vs. consume at current level. The results are illustrated in Figure 2.

We ran a logistic regression on the resulting variable using the following factors of interest: Social distance (in-group vs. out-group), Identification (identified vs. unidentified), and their interaction. But we also entered the following factors: Collection period (first vs. second), Perceived importance of feedback, Perceived similarity between participants' own household and referent household, Number of people in the participant's household, and Participant's perception of how their energy consumption really compares to that of their neighbors.

We first focused on the main variables of interest: Social distance, Identification, and their interaction. Social distance exerted an influence, Wald(1) = 6.34, *p* = 0.012, β = −1.08, Nagelkerke's *R*^2^ = 0.015. Overall, a greater percentage of participants stated that they would reduce their energy consumption level when the referent was in-group (50.7%) than when it was out-group (40.3%). Identification also exerted an influence, Wald(1) = 3.84, *p* = 0.050, β = −0.85, Nagelkerke's *R*^2^ = 0.004. Overall, a greater percentage of participants stated that they would reduce their energy consumption level when the referent was unidentified (48.2%) than when it was identified (42.8%). These main effects were qualified by a significant interaction, Wald(1) = 7.1, *p* = 0.008, β = 1.63, Nagelkerke's *R*^2^ = 0.018. These effects were carried by the very large influence that the In-group—Unidentified condition had in decreasing energy consumption (about 60%) vs. the other groups (all close to 40%), as shown in Figure 2.

Turning to the remaining factors, only the rated importance of the information had a statistically significant influence: Wald(1) = 51.7, *p* < 0.001, β = 1.0, Nagelkerke's *R*^2^ = 0.381. Perhaps unsurprisingly, the more participants perceived the feedback as relevant, the more they intended to decrease their energy consumption.

### Self-rated intention to modify consumption: Amount

We then focused on the percentage by which the participants intended to decrease their energy consumption (for the participants who selected to leave their consumption level unmodified, we inserted zeros). Dovetailing with the results from choice, the condition in which participants were willing to decrease consumption by the greatest amount was the In-group—Unidentified (*M* = 7.17%). We analyzed the data using a 2 (Social Distance) × 2 (Identification) ANOVA, and we included as covariates the four factors used in the previous analysis. There was no main effect of Social distance [*F*_(1, 268)_ = 0.23, *p* = 0.632, η_*p*_^2^ = 0.001] or Identification [*F*_(1, 268)_ = 0.15, *p* = 0.697, η_*p*_^2^ = 0.001]. However, once again, we found a significant interaction, *F*_(1, 268)_ = 8.31, *p* = 0.004, η_*p*_^2^ = 0.03. As was the case with choice, the only covariate that had a statistically significant influence was the perceived importance of the feedback, *F*_(1, 268)_ = 61.51, *p* < 0.001, η_*p*_^2^ = 0.19. These results are described in **Figure 4**.

Inspection of the data revealed that participants anchored their judgments on the suggested 10% decrease. Out of all participants stating that they intend to decrease their energy consumption, the majority (54%) intended to decrease it by exactly 10%. We will return to this finding in the General Discussion.

### Self-rated intention to modify consumption: Saving strategies

**Figure 3:**
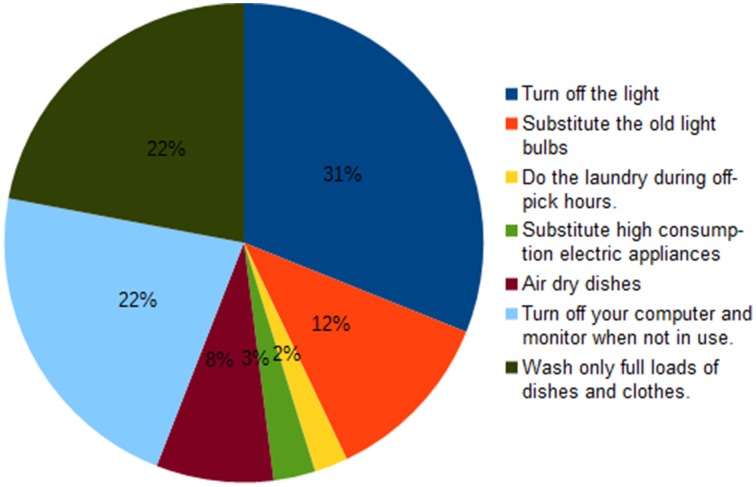
**A pie chart indicating the percentage of times that a saving strategy was chosen**.

A subset of the participants (*N* = 118) indicated the three ways by which they intended to save energy. The results are illustrated in Figure 3. The most chosen option was “Turn off the light when you exit the room” (31%), while the second and third most chosen options were “Turn off your computer and monitor when not in use” (22%) and “Wash only full loads of dishes and clothes” (22%). These data indicate that there is a certain degree of consistency across participants in their preferences about how to save energy.

## Summary

We examined whether the effect of comparative feedback (a typical household consumes 10% less) on self-rated intentions to modify energy consumption is moderated by information concerning the “typical household”: whether it is located in the same vs. a different neighborhood, and whether its members are identified by names, age, and a photograph vs. they remain unidentified. It was, but not in the way we had anticipated. The most successful intervention was the one where the referent household was from the same neighborhood and its members were unidentified (In-group—Unidentified).

## Control conditions

The analyses presented above lack a suitable control condition, a baseline. Theoretically, the self-rated intention to decrease energy consumption might be even higher if no feedback or just statistical feedback is given. To examine these possibilities, during the second collection period we gathered data from two control conditions: Statistical Feedback and No Feedback. Participants in the Statistical Feedback condition were informed that: “Your energy consumption exceeded the average household consumption level by 10%.” Participants in the No Feedback condition received no information about others' energy consumption levels. Subsequently, participants were asked to decide whether they intended to increase, keep constant, or decrease their energy consumption level (as in the experimental conditions, no one chose to increase energy consumption). In case they decided to change their consumption level, they had to indicate by how much (%). Because the control conditions offered no information or very abstract information about a referent household, we did not collect perceived similarity ratings between the participants' household and the referent household, or importance ratings of the feedback. Below, we compare the findings of the four experimental conditions to those of the control conditions.

## Results

### Self-rated intention to modify consumption: Choice

First, we compared the six conditions with a logistic regression, specifying the No Feedback condition as the baseline condition. In essence, this analysis examines the extent to which providing comparative feedback (social or statistical) promotes energy saving. As anticipated, feedback influenced the decision to save energy Wald(5) = 11.76, *p* = 0.038, Nagelkerke's *R*^2^ = 0.49. Overall, a higher percentage of participants chose to reduce their energy consumption in the Feedback conditions than in the No Feedback condition. However, out of the five comparisons, only the comparison between the In-group—Unidentified condition (*M* = 59.4%) and the No Feedback condition (*M* = 25%) was statistically significant [χ^2^ (1, *N* = 97) = 9.44; *p* = 0.002, φ = 0.31].

Subsequently, we tested whether the In-group—Unidentified condition was more effective than all the other feedback conditions. To this end, we compared it against a pooled condition that includes all other feedback conditions (for a similar analysis, see Goldstein et al., 2008, Exp. 2). It was (59.4% vs. 41.4%), χ^2^ (1, *N* = 306) = 7.04; *p* = 0.008, φ = 0.15. The results about choices are illustrated in Figure 2.

### Self-rated intention to modify consumption: Amount

**Figure 4:**
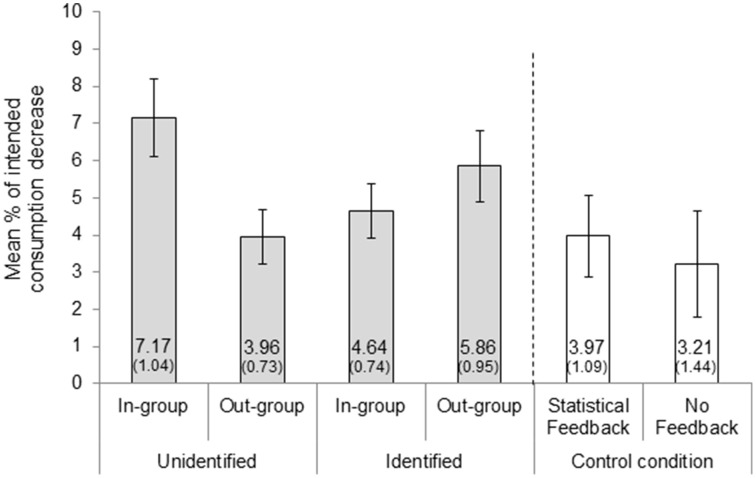
**Mean (SE) amount of intended consumption decrease by condition**. Error bars indicate the standard error of the mean. The number of participants in each group were as follows: In-Group Unidentified (*n* = 69), Out-Group Unidentified (*n* = 70), In-Group Identified (*n* = 69), Out-group Identified (*n* = 69), Statistical Feedback (*n* = 29), and No Feedback (*n* = 28).

Analyses of the specific amount by which participants were intending to decrease energy consumption, provided similar results (see Figure 4). We analyzed these data by means of a One-Way ANOVA. The mean amount of energy saving was influenced by condition, *F*_(5, 328)_ = 2.24, *p* = 0.050, η_*p*_^2^ = 0.03, with the In-group—Unidentified condition registering the highest amount of intended saving (*M* = 7.17%) and the No Feedback condition the lowest (*M* = 3.21%). Furthermore, participants in the In-group—Unidentified condition planned a significantly higher amount of intended saving (*M* = 7.17%) than all the other feedback conditions pooled together (*M* = 4.71%), *t*_(304)_ = −2.52; *p* = 0.012, Cohen's *d* = 0.32.

## Summary

As anticipated, providing feedback (either social or statistical) vs. not providing feedback increased both the percentage of choices to decrease energy consumption, as well as the amount of planned energy saving. Furthermore, from all types of feedback, the one concerning the In-group—Unidentified household promoted the highest energy consumption savings.

## General discussion

Findings in social psychology research have been used to create nudging techniques (Thaler and Sunstein, 2008). An effective technique to save energy is providing social feedback about *what* comparable others do (e.g., Goldstein et al., 2008). Here, we asked whether we could further sharpen this nudge. Building on research on the identified victim effect (e.g., Small and Loewenstein, 2003) we manipulated *who* the comparable others are on two dimensions: whether they came from the same vs. a different group, and whether they were identified vs. unidentified. We also included two control groups: a statistical feedback group and a no feedback group. In line with previous research, we found that feedback (vs. no feedback) increased both the intention to diminish energy consumption and the amount of consumption decrease. Importantly, one particular type of comparative feedback, the one concerning a household from the same neighborhood (in-group) but with no identifying details (unidentified), was the most effective.

This result is surprising. Most previous research suggests that people are more willing to help identified rather than unidentified individuals from one's in-group (e.g., Small and Loewenstein, 2003; Kogut and Ritov, 2005a,b; Slovic, 2007; see Introduction). However, recent studies have shown that under certain conditions this preference may reverse. One such case is when one's group is perceived as particularly homogeneous or cohesive (it has a high degree of “we-ness”; Bollen and Hoyle, 1990). In such circumstances, an individual might identify more with a generic group member than with an identified group member (see also Turner et al., 1987). For example, Ritov and Kogut (under review), (Study 2) conducted a second dictator game study. As in their Study 1, before playing the game participants were assigned into two groups supposedly on the basis of their artistic preferences. However, in Study 2 participants played a group game before playing the dictator game, in which they had to identify (as a group) as many characters as possible in a big poster. The purpose of this game was to increase group cohesiveness. Contrary to Study 1, in Study 2 “dictators” allocated significantly more money to in-group unidentified members (5.4 shekels) than to in-group identified members (3.8 shekels).

Returning to the present study, it could be that Israeli students perceived households from their neighborhood to be a highly cohesive category. One reason to expect this, is that the target city, Jerusalem, includes very diverse neighborhoods. For example, the Old City is roughly divided in the Jewish, Christian, Muslim, and Armenian Quarters. Research has shown that inter-group conflict increases the perception of cohesiveness with one's in-group (Ritov and Kogut, 2011). In a highly cohesive category, members of the category may perceive an unidentified, prototypical in-group member as more similar to themselves than an identified individual member. The effect of cohesiveness on perceived psychological distance may thus be at the source of the observed reversal of other-identifiability effect.

Another explanation for the reversal of the other-identifiability effect concerns the information provided in the identified condition, and specifically the photograph (see Figure 1). Participants might have found it curious that the energy company sent them a letter with a picture and names of other consumers (in other experimental contexts, such as donations, providing such details is unsurprising). Other forms of manipulations might have been more natural in the present experimental context. Our decision to use pictures and detailed descriptions was aimed at maximizing the emotional vividness of the identified conditions. However, future research could manipulate identifiability through other means.

Interestingly, if we consider all groups where feedback was provided, the majority of participants (53%) who decided to decrease their energy consumption opted for a 10% reduction, the exact amount required to match the norm. Although inferences from intentions to behaviors call for cautiousness (e.g., see Sheeran, 2002), previous field research has shown that comparative feedback makes participants change their actual consumption level (up or down) in the direction of the norm (e.g., Schultz et al., 2007). So, if intentions translate to behaviors, and if people have some sense of what it means to modify their energy consumption by a given amount, this could provide further means to nudge people toward energy savings.

The present findings carry potential benefits for various stakeholders. If such interventions prove successful, households could save money^2^. At a governmental level, a state that reduces energy consumption depends less from foreign energy supply, which in turn has strong economic and political advantages. Finally, a reduction in energy consumption can improve the well-being of future generations: saving energy implies a lower consumption of non-renewable resources (e.g., oil, coal) and a less polluted environment. At a policy level, nudge strategies utilizing comparative feedback are one of the several instruments that the national authorities have to increase energy efficiency. From a legal perspective, consumers have the right to have easy access to information about their actual consumption levels but also to complementary information, which refers “to the past consumption of an average final consumer or a target consumer belonging to the same category” [European Parliament and Council, 2012; Art.9, paragraph 7(e), Legislative Decree, July 4 2014, n. 102]. The aim of the Legislator is to facilitate such comparative evaluations for the final consumer. But, which is the optimal level of description to achieve this? This is not an easy question to answer. Previous studies (Kahneman et al., 1999; Bonini et al., 2008) show that, several elements influence how people categorize and interpret information during a comparative evaluation. Coherently with those results, the present findings suggest that subtle differences in the way the comparative consumer is described might yield strong differences in the willingness to reduce energy consumption.

As denoted by the subheading of our article, “A preliminary study, ” the present findings should be considered as a starting point. Their generalizability is limited for two reasons. First, they were based on Israeli university students. Future research could focus on different types of residential consumers, and from diverse geographical areas. This is important because research suggests not only that there are differences in energy consumption behaviors between cultures (Wilhite et al., 1996), but also within a given culture. For example, a recent study by Costa and Kahn (2013) on the influence of descriptive norms showed that, energy saving interventions in the US were more effective with registered liberals than with registered conservatives. Future studies, for example, could examine variables such as political orientation, and wealth. Second, the current study measured the intention to modify energy consumption rather than actual behavior. Ultimately, the effectiveness of the suggested intervention should be assessed by field studies.

In conclusion, we found that in a comparative social feedback it is not only important to know *what* others do, but also *who* these others are. Although, the present findings are preliminary, if supported, they would suggest a simple, cost-effective nudging technique to reduce people's energy consumption levels. Future studies should also investigate whether the In-group—Unidentified condition would always promote the highest compliance rate. We surmise that in certain cases, such as when group cohesiveness or homogeneity is low, social comparison with an In-group—Identified member might prove more efficient.

### Conflict of interest statement

The authors declare that the research was conducted in the absence of any commercial or financial relationships that could be construed as a potential conflict of interest.

## Footnotes

^1^A typical university student in Israel lives in a shared flat with other students. This should be especially true for most participants in our sample for two reasons: (1) about half of the them stated that, they lived on their own or with a single other person, (2) their mean age was 25.4, at which age most students do not live in their parents' home. Critically, students living in shared flats are responsible for paying their utility bills.

^2^Data from the World Energy Council (2014) indicate that the per household residential electricity consumption in Israel in 2013 was about 6400 kWh, with a price of.15 US dollar/kWh. These figures, applied to our findings, would translate in the following annual saving: $69 for the most successful intervention (in-group unidentified), compared to $45 for the other feedback conditions pooled together. These estimates, which should be used with caution, give a general idea of the potential economic impact of these *nudge* strategies.
