# Supplementary material for: To make people save energy tell them what others do but also who they are: a preliminary study
Source: Front Psychol. 2015 Aug 28;6:1287. doi: 10.3389/fpsyg.2015.01287 (PMC4551825; doi:10.3389/fpsyg.2015.01287)
Supplement: Supplementary file 1 [file DataSheet1.DOCX]

**Appendix A**

This appendix reports the instructions for the four experimental conditions and the two control conditions. Please note that the original instructions were in Hebrew, here we report an English translation. For the first condition, *Ingroup – Unidentified*, we present all the questions that we asked participants. For the following conditions, we just focus on the text that we manipulated (we highlight this text in italics).

*Ingroup – Unidentified condition*

Imagine that the letter containing your energy bill has arrived. You open it and notice that together with your energy bill there is also *a statement comparing your latest consumption level to the average consumption level of a typical apartment from your neighborhood (that is, an apartment where three students live).* The statement notes that:

Your energy consumption exceeded the typical apartment consumption of *your neighborhood* by 10%.

In light of this statement what do you plan to do? Please tick the option that applies below. If you select option 1 or 3, please specify also the appropriate level.

1. I plan to increase my energy consumption by approximately _____ %
2. I do not plan to either increase or decrease my energy consumption.
3. I plan to decrease my energy consumption by approximately _____ %

If you selected option 3 (decrease your energy consumption level), please specify the means by which you aim to achieve this by ticking up to three statements from the list below. Next to each of these statements, please indicate how much you are willing to actually implement these solutions: “1” = most likely to implement; “2” = second most likely to implement; “3” = third most likely to implement.

- Turn off the light when you exit the room. ____
- Substitute the old light bulbs in your house with low consumption ones. ____
- Do the laundry during off-pick hours. ____
- Substitute high consumption electric appliances (e.g. dishwashers, irons) with more energy efficient models. ____
- Air dry dishes instead of using your dishwasher’s drying cycle. ____
- Turn off your computer and monitor when not in use. ____
- Wash only full loads of dishes and clothes. ____

Questionnaire, Part 2.

- Including yourself, how many people live in your apartment? (Answer “1” if you live alone; “2” if you live with just one other person; etc.) _____
- To what extent do you consider important (for your energy consumption choices) the information given above about the typical apartment?

| 0 | 1 | 2 | 3 | 4 | 5 | 6 |
| --- | --- | --- | --- | --- | --- | --- |
| Not important at all |  |  | Quite important |  |  | Very important |

- To what extent do you feel that the place where you live is similar to the typical apartment in your neighborhood (that is, an apartment where three students live)?

| 0 | 1 | 2 | 3 | 4 | 5 | 6 |
| --- | --- | --- | --- | --- | --- | --- |
| Not similar at all |  |  | Quite similar |  |  | Very similar |

- How does your actual energy consumption level compare to the consumption level of other apartments in your neighborhood that have a similar composition to yours (that is, other apartments with the same number of individuals)? Please tick a number below:

| -3 | -2 | -1 | 0 | +1 | +2 | +3 |
| --- | --- | --- | --- | --- | --- | --- |
| My consumption is much lower |  |  | My consumption is similar |  |  | My consumption is much higher |

- In which neighborhood do you live? ____________________

*Ingroup – Identified condition*

Imagine that the letter containing your energy bill has arrived. You open it and notice that together with your energy bill there is also *a statement comparing your latest consumption level to the average consumption level of a typical apartment from your neighborhood (for instance, the apartment where the following three students live: Mary, 23 years old, John, 25 years old, and Elizabeth, 24 years old; see below one of their pictures).*

*[FIGURE 1]*

The statement notes that:

Your energy consumption exceeded the typical apartment consumption of *your neighborhood* by 10%.

*Outgroup – Unidentified condition*

Imagine that the letter containing your energy bill has arrived. You open it and notice that together with your energy bill there is also *a statement comparing your latest consumption level to the average consumption level of a typical apartment in Haifa (that is, an apartment where three students live).* The statement notes that:

Your energy consumption exceeded the typical apartment consumption of *a neighborhood in Haifa* by 10%.

*Outgroup – Identified condition*

Imagine that the letter containing your energy bill has arrived. You open it and notice that together with your energy bill there is also *a statement comparing your latest consumption level to the average consumption level of a typical apartment in Haifa (for instance, the apartment where the following three students live: Mary, 23 years old, John, 25 years old, and Elizabeth, 24 years old; see below one of their pictures*).

*[FIGURE 1]*

The statement notes that:

Your energy consumption exceeded the typical apartment consumption of *a neighborhood in Haifa* by 10%.

*Control with reference condition*

Imagine that the letter containing your energy bill has arrived. *You open it and notice that together with your energy bill there is also a statement comparing your latest consumption level to the average consumption level.* The statement notes that:

Your energy consumption exceeded the average household consumption level by 10%.

*Control without reference condition*

[Participants had to respond directly to the following questions]

Do you plan to change you habitual energy consumption level?

Please tick the option that applies below. If you select option 1 or 3, please specify also the appropriate level.

1. I plan to increase my energy consumption by approximately _____ %
2. I do not plan to either increase or decrease my energy consumption.
3. I plan to decrease my energy consumption by approximately _____ %
